# ^68^Ga-PSMA-11 in Staging of Unfavorable Intermediate- and High-Risk Prostate Cancer Reduces Indication for Noncurative Prostatectomy: A Prospective, Multicenter, IAEA Study

**DOI:** 10.2967/jnumed.125.270537

**Published:** 2026-01

**Authors:** Juliano J. Cerci, Stefano Fanti, Enrique E. Lobato, Rakesh Kumar, Jolanta Kunikowska, Akram Al-Ibraheem, Maisarah Nasir, Francisca Redondo Moneda, Osvaldo Garcia, Mohamad Haidar, Fuad Novruzov, Ozlem Kucuk, Umut Elboga, Murilo de Almeida Luz, Diana Paez

**Affiliations:** 1 Quanta Diagnostico e Terapia, Curitiba, Brazil;; 2 IRCCS Azienda Ospedaliero–Universitaria di Bologna, Bologna, Italy;; 3 Division of Human Health, International Atomic Energy Agency, Vienna, Austria;; 4 All India Institute of Medical Sciences, New Delhi, India;; 5 Nuclear Medicine Department, Medical University of Warsaw, Warsaw, Poland;; 6 King Hussein Cancer Center, Amman, Jordan;; 7 Institute Kanser Negara, Putrajaya, Malaysia;; 8 Asistencial Sotero del Río, Santiago Clínica Andes Salud, Puerto Montt, Chile;; 9 Instituto Nacional de Cancerologia, Tlalpan, Mexico;; 10 American University of Beirut Medical Center, Beirut, Lebanon;; 11 Nuclear Medicine Department, National Centre of Oncology, Baku, Azerbaijan;; 12 Ankara University, Ankara, Turkey;; 13 University of Gaziantep, Gaziantep, Turkey; and; 14 Mount Sinai Health System, New York, New York

**Keywords:** GU oncology, prostate cancer, PET/CT, PSMA, initial staging

## Abstract

Accurate staging of unfavorable intermediate- or high-risk prostate cancer (PCa) is essential for treatment decisions. Conventional imaging often fails to detect lymph node, bone, and visceral metastases, and for this purpose ^68^Ga-prostate-specific membrane antigen (PSMA)–11 PET/CT is clinically used. This prospective, multicenter, International Atomic Energy Agency–supported trial evaluated the accuracy of ^68^Ga-PSMA-11 PET/CT for initial staging compared with MRI and histopathology and the impact of ^68^Ga-PSMA-11 PET/CT on determining surgical eligibility. **Methods:** In a prospective, international study supported by the International Atomic Energy Agency, 775 patients with high-risk or unfavorable intermediate-risk PCa from 12 centers across 11 countries—including low-, middle-, and high-income settings, scheduled for radical prostatectomy based on conventional imaging (including bone scanning and pelvic MRI) underwent ^68^Ga-PSMA-11 PET/CT before treatment. PET and MRI findings were compared with radical prostatectomy histopathology, and the impact of PET on radical prostatectomy was assessed. **Results:** ^68^Ga-PSMA-11 PET/CT detected metastatic disease (M1) in 20.4% of cases, altering management and preventing prostatectomy in 24.0%. The accuracy for seminal vesicle invasion was 90.1% for ^68^Ga-PSMA-11 PET/CT versus 57.3% for MRI, and for lymph node metastases it was 91.1% for ^68^Ga-PSMA-11 PET/CT versus 69.7% for MRI. In 13.1% of patients (78/593), there were discordant results between ^68^Ga-PSMA-11 PET/CT and histopathology. ^68^Ga-PSMA-11 PET/CT had false-negative lymph node findings in 8.6% of cases, with the most clinically significant being 4.5% of patients incorrectly staged as N0. False-positive lymph node findings at ^68^Ga-PSMA-11 PET/CT occurred in 4.5% of patients. **Conclusion:** ^68^Ga-PSMA-11 PET/CT significantly improves staging accuracy, reducing the indication for prostatectomy and impacting treatment decisions. These findings, from a broad international cohort including low-, middle-, and high-income countries, support the global adoption of ^68^Ga-PSMA-11 PET/CT into standard staging protocols for high-risk PCa.

## Visual Abstract

**Figure:**
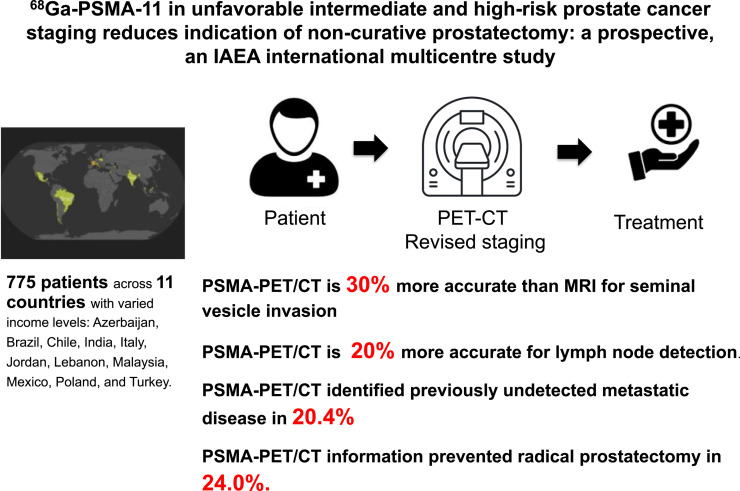

Accurate staging of prostate cancer (PCa) is essential for guiding treatment decisions and useful for predicting patient outcomes. Conventional imaging modalities, such as CT and bone scanning, have long been used to assess local and distant disease burden, and more recently MRI has been used as well. However, these techniques have several limitations, especially low sensitivity in detecting involvement of lymph nodes and metastatic disease, resulting in a suboptimal impact on therapeutic strategies (1,2).

Over the past decade, ^68^Ga-prostate-specific membrane antigen (PSMA) PET/CT has emerged as a more sensitive and specific imaging modality for assessing PCa burden. PSMA is highly expressed in PCa cells, and radiolabeled tracers targeting this antigen allow for the detection of metastases that may not be visualized with conventional imaging (3–5).

The increasing global use of ^68^Ga-PSMA-11 PET/CT has prompted further evaluation of its diagnostic accuracy and prognostic value, and several papers have confirmed the usefulness of such an approach. However, several aspects remain unclear, including the ultimate accuracy of ^68^Ga-PSMA-11 PET/CT and the impact on treatment decisions. Supported by the International Atomic Energy Agency (IAEA) in a multicenter worldwide trial with low-, intermediate-, and high-income countries, this study aimed to characterize the extent of PCa (local, nodal, and metastatic involvement) using ^68^Ga-PSMA-11 PET/CT; compare the diagnostic performance of ^68^Ga-PSMA-11 PET/CT and MRI, using as a gold standard the radical prostatectomy histopathology findings; and assess the impact of ^68^Ga-PSMA-11 PET/CT on treatment decision-making, specifically changes in surgical indication.

## MATERIALS AND METHODS

### Study Design and Population

This prospective, multicenter, internationally designed trial enrolled newly diagnosed, biopsy-confirmed, treatment-naïve, high-risk or unfavorable intermediate-risk PCa patients who were candidates for radical prostatectomy as staged to be nonmetastatic on the basis of conventional imaging (CT, bone scanning, and abdominopelvic MRI).

Standard forms for data registration were developed and agreed on among the investigators. Data were collected for ^68^Ga-PSMA-11 PET/CT positivity rate, localization of positive findings, and impact on patient management. All centers obtained local ethical clearance for prospective recruitment of patients and data collection, according to national regulations. All subjects signed an informed consent form.

The study involved 12 centers from 11 countries, totalizing 775 research participants: Azerbaijan, 29 patients; Brazil, 180; Chile, 38; India, 160; Italy, 99; Jordan, 64; Lebanon, 30; Malaysia, 43; Mexico, 31; Poland, 69; and Turkey, 32.

All patients underwent ^68^Ga-PSMA-11 PET/CT for primary staging before definitive treatment decisions were finalized.

Studies were performed accordingly to existing procedural international guidelines (6). On the basis of procedure guidelines for PCa imaging, the findings were classified as either positive or negative regarding prostate, seminal vesicle, lymph node, and metastatic involvement.

Either analog or digital tomographs were allowed; true whole-body imaging, including lower limbs, contrast-enhanced CT obtained with application of a diuretic was also allowed. All centers had more than 5 y of experience reporting ^68^Ga-PSMA-11 PET/CT results.

### Imaging and Histopathologic Comparison

^68^Ga-PSMA-11 PET/CT and MRI findings were evaluated for seminal vesicle invasion, pelvic lymph node metastases (N1), and distant metastases (M1), including distant lymph nodes, bones, and visceral lesions.

The reported imaging findings were compared with histopathologic analysis of radical prostatectomy specimens in patients who proceeded with surgery. Sensitivity, specificity, positive predictive value, and negative predictive value were calculated for seminal vesicle invasion and lymph node involvement for both ^68^Ga-PSMA-11 PET/CT and MRI.

### Impact on Treatment Decisions

Pre– and post–^68^Ga-PSMA-11 PET/CT data regarding intention to treat and final therapeutic decision were prospectively collected to assess changes in the surgical indication and overall management.

## RESULTS

### Detection of Extent of Disease

The clinical characteristics of the patients are detailed in Table 1.

**TABLE 1.** Clinical Characteristics of Patients

Gleason score	*n*	%
3 + 4	102	13.2%
3 + 5	7	0.9%
4 + 3	231	29.8%
4 + 4	240	31.0%
4 + 5	123	15.9%
5 + 3	3	0.4%
5 + 4	42	5.4%
5 + 5	26	3.4%
ISUP		
2	73	9.4%
3	303	39.1%
4	280	36.1%
5	192	24.8%
High risk	555	71.6%
	Median	
Age (y)	67.35	
PSA (ng/mL)	15.8	

ISUP = International Society of Urological Pathology; PSA = prostate-specific antigen.

^68^Ga-PSMA-11 PET/CT detected metastatic disease (M1) in 20.4% (158/775) of patients; seminal vesicle invasion was detected in 37.9% (294/775); and pelvic lymph node involvement (N1) was detected in 43.5% (337/775).

Distant metastases (M1) were confirmed histologically in only 15 patients (<10% of patients with M1 disease) for reasons such as small lesions, poor location, and the aggressiveness of the procedure required.

### ^68^Ga-PSMA-11 PET/CT Worldwide

The centers were grouped by country income. High-income countries included Italy and Poland (168 patients); countries with upper middle income included Azerbaijan, Chile, Brazil, Jordan, Mexico, Malaysia, and Turkey (417 patients); and those with lower middle income included India and Lebanon (190 patients). There were no significant differences (*P* = 0.73) between positivity of ^68^Ga-PSMA-11 PET/CT N1 (47.3%, 43.6% and 38.7%) and M1 (24.2%, 19.9% and 17.8%) in patients with a lower middle, upper middle, and high income, respectively.

### Impact on Surgical Management

After ^68^Ga-PSMA-11 PET/CT, in 24.0% (186/775) of patients, the local multidisciplinary tumor boards decided not to proceed with the previous planned radical prostatectomy because of the detection of distant metastases or lymph node involvement. Instead of radical prostatectomy, 22 patients underwent radiotherapy plus androgen deprivation therapy (ADT), 124 underwent ADT alone, 14 underwent androgen receptor pathway inhibitors plus ADT, and 26 underwent chemotherapy plus ADT.

The local tumor boards also decided, on the basis of each center’s treatment protocol, that 20.2% (32/158) of ^68^Ga-PSMA-11 PET/CT–metastatic patients would undergo prostatectomy and ADT.

### Diagnostic Performance: ^68^Ga-PSMA-11 PET/CT Versus MRI

Among the 76.5% (593/775) of patients who underwent radical prostatectomy, ^68^Ga-PSMA-11 PET/CT findings were compared with histopathologic results (Table 2). ^68^Ga-PSMA-11 PET/CT was more than 30% more accurate than MRI for seminal vesicle invasion and more than 20% more accurate for lymph node detection.

**TABLE 2.** MRI and ^68^Ga-PSMA-11 PET/CT Findings Compared with Histopathologic Results

Lesion type	Modality	Sensitivity	Specificity	PPV	NPV	Accuracy
Seminal vesicle invasion	PSMA PET/CT	85.1%	93.0%	83.7%	93.7%	90.7%
	MRI	29.4%	88.6%	74.3%	52.9%	57.3%
Pelvic lymph nodes	PSMA PET/CT	87.5%	93.5%	90.0%	91.8%	91.1%
	MRI	23.1%	93.0%	62.2%	70.1%	69.7%

PPV = positive predictive value; NPV = negative predictive value.

### Discordance Between ^68^Ga-PSMA-11 PET/CT and Histopathology

In 13.1% (78/593) of cases, there were discrepancies between PET/CT findings and histopathology regarding lymph node involvement. Twenty-one patients had positive lymph node findings on ^68^Ga-PSMA-11 PET/CT but no lymph node metastases on histopathology. Twenty-seven patients had negative lymph node findings on ^68^Ga-PSMA-11 PET/CT but lymph node metastases on histopathology. Thirty patients had fewer positive lymph node chains on PET/CT than on histopathology.

## DISCUSSION

### Clinical Impact of ^68^Ga-PSMA-11 PET/CT on PCa Management

This multicenter trial demonstrated that ^68^Ga-PSMA-11 PET/CT significantly alters clinical management in high-risk PCa by providing higher accuracy in detecting metastases (7–10). Nearly 1 in 4 patients who initially were considered candidates for radical prostatectomy had their treatment plans altered after PET/CT staging, preventing a noncurative surgery in those with undetected metastatic disease. Even if histologic confirmation of distant metastases was performed in a limited number of patients, the multidisciplinary team–based decisions ensured appropriate management and reflected common clinical practice. A dedicated follow-up protocol is ongoing, and a separate survival-focused publication is under preparation.

The ProPSMA study, a multicenter, randomized phase 3 trial, evaluated the impact of ^68^Ga-PSMA-11 PET/CT imaging on the management of high-risk PCa patients. The study demonstrated that ^68^Ga-PSMA-11 PET/CT led to significant changes in treatment plans for 28% of patients, also in line with our results (11).

### Worldwide Applicability and Relevance

Cost and accessibility remain relevant challenges, particularly in low- and middle-income countries. A key finding of our study is that there were no statistically significant differences in ^68^Ga-PSMA-11 PET/CT performance across different income categories among participating countries. This finding underscores that, despite significant variations in health care systems and resources worldwide, nations can consistently deliver high-quality ^68^Ga-PSMA-11 PET/CT studies when conducted in appropriate medical centers regardless of the country’s income level. A similar result of PSMA reproducibility use worldwide has already been shown in a different scenario of biochemical relapse (12).

A dedicated cost-effectiveness analysis within this IAEA-supported cohort is under way.

### ^68^Ga-PSMA-11 PET/CT Versus MRI: Diagnostic Superiority

**FIGURE 1.**
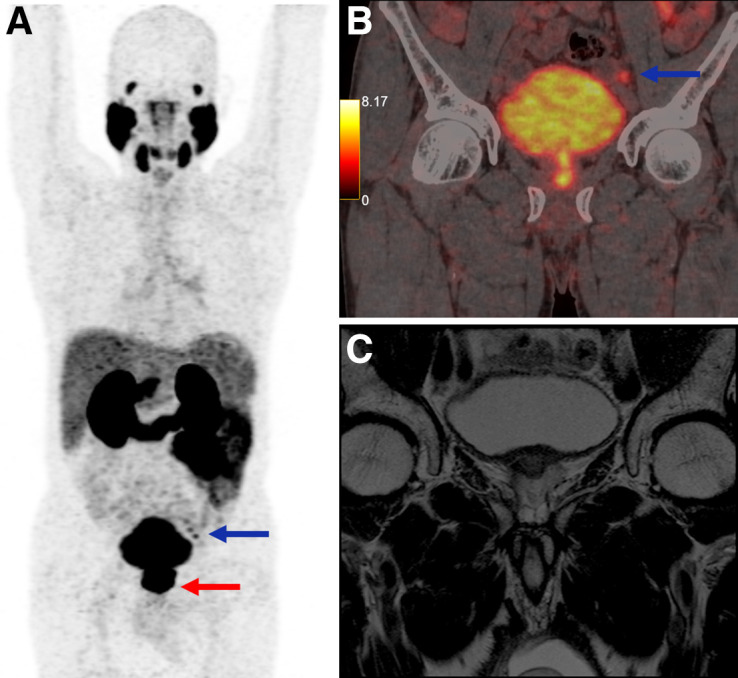
A 72-y-old man presenting for initial staging of PCa. (A) ^68^Ga-PSMA-11 PET/CT maximum-intensity projection shows PSMA-expressing lesion involving prostate (red arrow), with PSMA avidity in left external iliac lymph nodes (blue arrow). (B) PET/CT coronal image shows 1 lymph node involved (blue arrow). (C) However, MRI at same topography shows no lymph node involvement. Pathology analysis confirmed ^68^Ga-PSMA-11 PET/CT–positive result.

**FIGURE 2.**
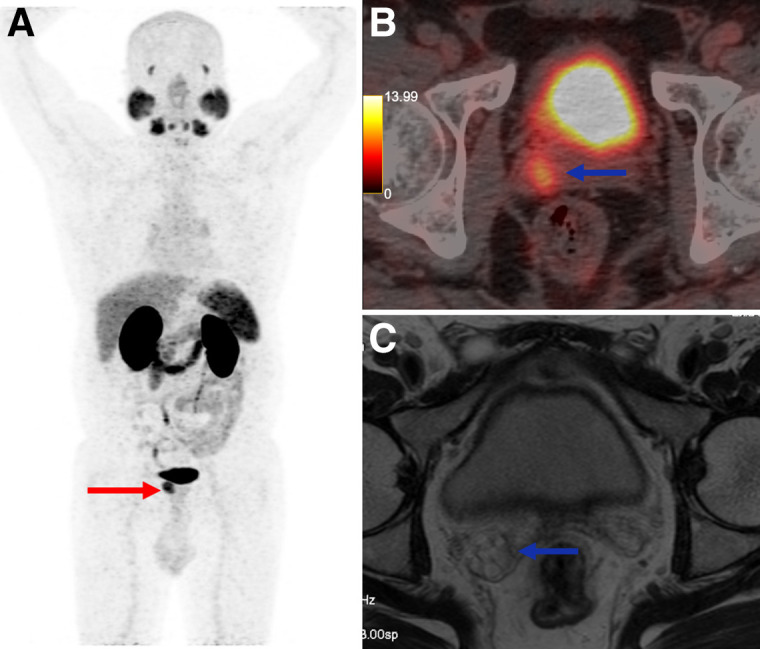
A 55-y-old man presenting for initial staging of PCa. (A) ^68^Ga-PSMA-11 PET/CT maximum-intensity projection shows PSMA-expressing lesion involving prostate (red arrow), with PSMA avidity in right seminal vesicle invasion (blue arrow). (B) PET/CT axial image shows right seminal vesicle invasion (blue arrow). (C) However, MRI at same topography shows no seminal vesicle invasion. Pathology analysis confirmed ^68^Ga-PSMA-11 PET/CT–positive results.

^68^Ga-PSMA-11 PET/CT demonstrated nearly 3 times higher sensitivity for seminal vesicle invasion (85.1% vs. 29.4%) and almost 4 times higher sensitivity for lymph node metastases (87.5% vs. 23.1%) than did MRI (Figs. 1 and 2). These findings reinforce the limited ability of MRI to detect seminal vesicle invasion and N1 disease, supporting the routine integration of ^68^Ga-PSMA-11 PET/CT in high-risk PCa staging (9).

Hope et al. conducted a metaanalysis evaluating the accuracy of ^68^Ga-PSMA-11 PET/CT for detecting pelvic lymph node metastases (N1) in PCa patients (6). Their findings confirm the superiority of ^68^Ga-PSMA-11 PET/CT over conventional imaging (CT or MRI) for nodal staging, supporting its role in refining treatment strategies, even if the pooled sensitivity of 74% was lower than the sensitivity we found, 87.5%. This considerable difference between sensitivities might be related to the metaanalysis inclusion of heterogeneous patient populations, imaging protocols, and reference standards across the analyzed studies, which may have contributed to variability in diagnostic accuracy. Also, in the metaanalysis there was a lack of standardized histopathologic validation, as not all patients underwent lymph node dissection. Our trial, although including patients from various centers, standardized the analysis of results.

### Limitations of ^68^Ga-PSMA-11 PET/CT

Despite its high accuracy, ^68^Ga-PSMA-11 PET/CT had false-negative lymph node findings in 8.6% of cases; 4.5% of these were more concerning because they were reported as N0 on PSMA PET, mostly likely because of microscopic metastases below PET resolution. The other 4.1% were N1 on PSMA PET, but histology demonstrated more involved lymph node chains than did PSMA PET. In the metaanalysis by Corfield et al. (13), ^68^Ga-PSMA PET/CT had a pooled result of 39% of patients presenting metastatic lymph nodes on histology but false-negative PSMA PET/CT results. This discrepancy is difficult to explain in view of the expertise of the surgeons, pathologists, and nuclear medicine physicians of our study.

Another 3.5% were considered to have false-positive ^68^Ga-PSMA-11 PET/CT lymph node findings. These could be related to inflammation or benign lymph node activity, which may occasionally mimic malignancy, but could also indicate malignant lymph nodes that were not accessed on radical prostatectomy. Further refinements in imaging interpretation and artificial intelligence–assisted analysis may help reduce these occurrences.

## CONCLUSION

This multicenter, IAEA-supported trial confirmed that ^68^Ga-PSMA-11 PET/CT is superior to MRI for primary staging of high-risk PCa. The high accuracy, sensitivity, and specificity in detecting seminal vesicle invasion, lymph node involvement, and distant metastases make ^68^Ga-PSMA-11 PET/CT the preferred imaging modality for treatment planning.

^68^Ga-PSMA-11 PET/CT altered management in nearly one quarter of patients, reducing the indication for noncurative prostatectomy in those with metastases not detected on conventional imaging. These findings, from a broad international cohort including low-, middle-, and high-income countries, support the incorporation of ^68^Ga-PSMA-11 PET/CT into standard staging for high-risk PCa, particularly in patients for whom radical prostatectomy is being considered.

## DISCLOSURE

This research was partially funded by IAEA. No other potential conflict of interest relevant to this article was reported.

## Notes

KEY POINTS **QUESTION:** Does ^68^Ga-PSMA-11 PET/CT improve initial staging detection of metastatic disease and influence surgical eligibility in patients with high- or unfavorable intermediate-risk PCa in a worldwide scenario? **PERTINENT FINDINGS:** In this prospective, multicenter, IAEA-supported trial including 775 patients across 11 countries with varied income levels, ^68^Ga-PSMA-11 PET/CT identified previously undetected metastatic disease in 20.4% and prevented radical prostatectomy in 24.0%. Compared with MRI, it demonstrated significantly higher sensitivity for detecting seminal vesicle invasion and lymph node metastases. **IMPLICATIONS FOR PATIENT CARE:** Incorporation of ^68^Ga-PSMA-11 PET/CT into standard staging protocols may improve treatment selection and avoid radical prostatectomy in patients with high-risk PCa not only from high-income countries but also from low- and middle-income countries.
